# Tungsten Oxide Coated Liquid Metal Electrodes via Galvanic Replacement as Heavy Metal Ion Sensors

**DOI:** 10.3390/s24020416

**Published:** 2024-01-10

**Authors:** Sagar Bhagwat, Leonhard Hambitzer, Richard Prediger, Pang Zhu, Ahmed Hamza, Sophia K. Kilian, Sebastian Kluck, Pegah Pezeshkpour, Frederik Kotz-Helmer, Bastian E. Rapp

**Affiliations:** 1 Laboratory of Process Technology, NeptunLab, Department of Microsystems Engineering (IMTEK), University of Freiburg, Georges-Köhler-Allee 103, 79110 Freiburg im Breisgau, Germany; sagar.bhagwat@neptunlab.org (S.B.); leonhard.hambitzer@neptunlab.org (L.H.); richard.prediger@neptunlab.org (R.P.); pang.zhu@neptunlab.org (P.Z.); ahmed.hamza@neptunlab.org (A.H.); sebastian.kluck@neptunlab.org (S.K.); pegah.pezeshkpour@neptunlab.org (P.P.); bastian.rapp@neptunlab.org (B.E.R.); 2 Hahn-Schickard, Georges-Köhler-Allee 103, 79110 Freiburg im Breisgau, Germany; sophia.kilian@hahn-schickard.de; 3 Freiburg Materials Research Center (FMF), University of Freiburg, Stefan-Meier-Straße 21, 79104 Freiburg im Breisgau, Germany; 4 FIT Freiburg Center of Interactive Materials and Bioinspired Technologies, University of Freiburg, Georges-Köhler-Allee 105, 79110 Freiburg im Breisgau, Germany

**Keywords:** liquid metals, galvanic replacement, heavy metal ions, sensor, photostructuring

## Abstract

Gallium liquid metals (LMs) like Galinstan and eutectic Gallium-Indium (EGaIn) have seen increasing applications in heavy metal ion (HMI) sensing, because of their ability to amalgamate with HMIs like lead, their high hydrogen potential, and their stable electrochemical window. Furthermore, coating LM droplets with nanopowders of tungsten oxide (WO) has shown enhancement in HMI sensing owing to intense electrical fields at the nanopowder-liquid–metal interface. However, most LM HMI sensors are droplet based, which show limitations in scalability and the homogeneity of the surface. A scalable approach that can be extended to LM electrodes is therefore highly desirable. In this work, we present, for the first time, WO-Galinstan HMI sensors fabricated via photolithography of a negative cavity, Galinstan brushing inside the cavity, lift-off, and galvanic replacement (GR) in a tungsten salt solution. Successful GR of Galinstan was verified using optical microscopy, SEM, EDX, XPS, and surface roughness measurements of the Galinstan electrodes. The fabricated WO-Galinstan electrodes demonstrated enhanced sensitivity in comparison with electrodes structured from pure Galinstan and detected lead at concentrations down to 0.1 mmol·L^−1^. This work paves the way for a new class of HMI sensors using GR of WO-Galinstan electrodes, with applications in microfluidics and MEMS for a toxic-free environment.

## 1. Introduction

Gallium liquid metals (LMs) like EGaIn (75% Ga, 25% In) and Galinstan (68.5% Ga, 21.5% In, and 10% Sn) have seen numerous applications in the past decade, owing to their negligible toxicity compared with mercury, water-like viscosity suited for microfluidics, exceptionally high surface tension (>500 mN·m^−1^), high electrical and thermal conductivity, and low vapor pressures (<10^−6^ bar), making them suitable for applications outside of a fume hood [1,2,3]. These properties have resulted in liquid metal-enabled microfluidic devices [4,5], pumps [6,7], actuators [8,9], droplet generators [10,11,12,13], flexible and reconfigurable electronics [14,15,16,17], antennas [18,19,20], soft robots [21], and sensors [22,23].

Among the many applications of LMs, Galinstan droplets have been used as working electrodes for heavy metal ion (HMI) sensors owing to their unique ability to amalgamate with various HMIs, such as lead (Pb^2+^), cadmium (Cd^2+^), copper (Cu^2+^), bismuth (Bi^3+^), and antimony (Sb^3+^); their high hydrogen potential (−0.63 V), which results in higher selectivity; and their good electrochemical stability [23,24]. HMIs are typically released from mining, vehicle exhausts, as battery manufacturing byproducts, and agricultural fertilizers, which tend to heavily pollute the environment with long-term life-threatening effects on human health, even in trace amounts [25]. In the recent decade, significant work has been performed towards detecting trace amounts of HMIs using LMs [25]. One promising approach is to utilize micro- and nanopowders like tungsten oxide (WO) to enhance the sensitivity of such HMI sensors, by increasing the effective surface area of the sensor, i.e., electrodes [26]. Sivan et al. showed 10 mmol·L^−1^ Pb^2+^ detection with Galinstan marbles (macro-droplets), with significant sensitivity enhancement for Galinstan marbles coated with 80 nm WO nanopowders [27]. They attributed this enhancement to the localized electrical fields at the nanoparticle/Galinstan/electrolyte triple-phase boundary. In a different approach, Zhang et al. transferred the Galinstan marbles to Galinstan/WO nanoparticles via sonication, which resulted in the detection of 100 ppb Pb^2+^ owing to the surface area-induced enhancement and the generation of a localized electrical field [28]. Similarly, EGaIn/Reduced Graphene Oxide (RGO) nanoscale particles prepared via sonication in solution showed enhanced sensitivities to detect 0.1 ppb of Cd^2+^, owing to an increase in the electrochemical area at the EGaIn/RGO/electrolyte interface [29]. However, the powder coating method and sonication-based dispersions are restricted to Galinstan marbles, with a high tendency towards forming very inhomogeneous surfaces. There are significant limitations in coating planar geometries like electrodes using LM droplets [27,30]. One common technique is stripping voltammetry as a favorable HMI sensing technique with trace amount detection limits, enhanced selectivity, and portability. Stripping voltammetry includes the critical selectivity step, which is the accumulation of HMIs via reduction on the working electrode surface, followed by stripping off by applying a voltage scan [25]. Improving this technique benefits from having working electrodes that are planar and enhance the accumulation of HMIs on their surface owing to the increased electrode effective surface area. Sensors for HMI detection based on screen-printed electrodes with a high sensitivity (0.43 nmol·L^−1^) and scalability have been presented [31,32]; however, a technique that provides a homogeneous coating, which is applicable to scalable planar electrodes from liquid metals is highly sought after in HMI detection.

Galvanic replacement (GR) is an established technique that enables the generation of functional coatings with tunable and controllable properties with respect to size, composition, shape, and structure, for applications like catalysis, sensors, and electronics [33,34]. It is based on a redox reaction between the metal ions of a sacrificial template with a lower reduction potential and the metal ions in solution with a higher reduction potential. This potential difference results in the reduction of metal ions in a solution phase and their deposition on the sacrificial template [35]. In recent years, GR of gallium liquid metals with various metal ions in solution has been successfully shown to allow the generation of controllable nanostructured surfaces for noble and non-noble metals, such as gold, platinum, silver, and copper; semiconductors, such as manganese and molybdenum oxide; and core-shell metal/metal oxides, such as WOx and VOx [36,37,38,39,40,41,42,43,44,45,46]. This mainly stems from the negative standard reduction potentials of −0.529 V (Ga^3+^/Ga^0^), −0.340 V (In^3+^/In^0^), and −0.138 V (Sn^2+^/Sn^0^), which readily undergo GR reactions with metal ions possessing higher standard reduction potentials, e.g., 0.799 V (Ag^+^/Ag^0^) [36]. Furthermore, GR is not limited only to droplets and can be conveniently extended to planar geometries like electrodes as key parts of a sensor. Fabricating a WO-coated Galinstan sensor using GR and a tungsten salt solution would allow for scalability, better control over the WO layer, and improved sensing capabilities of HMI like Pb^2+^ on Galinstan planar electrodes with an increased surface area.

In this work, we present, for the first time, WO-Galinstan electrodes fabricated via photolithography, Galinstan brushing, lift-off, and GR in a sodium metatungstate monohydrate (W salt) solution. We first confirmed successful GR on Galinstan electrodes by examining the effect of W salt concentrations (10, 50, 100, 200, and 400 mM) over 24 h, and further showed that 24 h treatment with 400 mM W salt solution is the optimal GR concentration. We support our findings using Scanning Electron Microscopy (SEM), Energy Dispersive X-ray spectroscopy (EDX), X-ray Photoelectron Spectroscopy (XPS), X-ray Diffraction (XRD), and White Light Interferometry (WLI) measurements. The presented WO-Galinstan electrodes have a detection limit of 0.1 mmol·L^−1^ Pb^2+^, and outperformed pure Galinstan electrodes with three times higher sensitivity. This work paves the way towards liquid metal electrodes using GR for HMI sensors in MEMS and microfluidics for environmental applications.

## 2. Materials and Methods

### 2.1. Materials

Galinstan was purchased and used as received from Strategic Elements (Deggendorf, Germany). Sodium metatungstate monohydrate was purchased from abcr GmbH (Karlsruhe, Germany). Distilled water was used as a solvent for preparing the salt solutions of different concentrations. A 24-well cell culture plate from Greiner Bio-One (Frickenhausen, Germany) was used for the galvanic replacement experiments. 2-propanol, acetone, ethanol, potassium chloride (KCl, ≥99.5%), and potassium nitrate were purchased from Carl Roth (Karlsruhe, Germany) and were used as received. Acetic acid (100%, glacial) and lead (II) acetate trihydrate were purchased from Sigma Aldrich (Darmstadt, Germany). Ammonium acetate was obtained from VWR (Darmstadt, Germany). Borosilicate glass slides (76 × 25 × 1 mm) were provided by Schott (Mainz, Germany). AZ 1518 positive photoresist and AZ 351B developer were purchased from MicroChemicals GmbH (Ulm, Germany). Platinum (0.3 mm diameter) and silver wire (0.25 mm diameter) were purchased from ChemPUR (Karlsruhe, Germany). Commercially available chlorine containing mold destroyer (45.9 g·L^−1^ Sodium Hypochlorite) of type Mellerud was purchased from OBI (Freiburg, Germany). Nitrogen gas was used from the in-house supply.

### 2.2. Methods

#### 2.2.1. Galvanic Replacement

An amount of 400 mM W salt solution was prepared as a stock solution using distilled water and diluted as necessary for GR. A 10 µL droplet of Galinstan was immersed in 1 mL W salt solutions of 10, 50, 100, 200, and 400 mM for a period of 24, 48, and 72 h. The resulting change in the droplet appearance was captured using optical microscopy images at 24, 48, and 72 h.

#### 2.2.2. Galinstan and WO-Galinstan Sensor Fabrication

We adapted the procedure described by Park et al. [47]. Briefly, glass slides were first rinsed with 2-propanol and dried using compressed nitrogen (N_2_). Around 1.5 mL of AZ 1518 photoresist was drop-casted and a 1.8 µm layer was spin-coated at 4000 rpm for 30 s, followed by a solvent prebaking step at 100 °C for 50 s according to the manufacturer. The designed and laser-printed electrode pattern (Koenen GmbH, Hohenbrunn, Germany) was placed on the spin-coated substrates and exposed at 415 nm for 45 s (exposure dose of 16 mJ·cm^−1^) using a mercury lamp of type Superlite S 04 (Lumatec, Oberhaching, Germany). The exposed slides were developed in AZ 351B for 15 s, rinsed with distilled water, dried with compressed N_2_, and post-baked for 1 min at 115 °C. The developed slides were further subjected to 2 min atmospheric plasma (Atto Plasmacleaner, Diener Electronic, Ebhausen, Germany) followed by drop-casting 10 µL Galinstan and using a glass vial to roller-blade it on the pattern. The Galinstan-coated slides were then left in an acetone bath for 30 min to lift-off the excess Galinstan and photoresist. An amount of 400 mM W salt solution was added on top of the Galinstan pattern and left to react for 24 h, followed by rinsing with distilled water and drying with compressed N_2_.

#### 2.2.3. Differential Pulse Stripping Voltammetry (DPSV) for Sensor Measurements

We adapted the procedure for fabricating a lab-made Ag-AgCl electrode as described by Barlag et al. [48]. In short, a mixture of agarose, potassium nitrate, and water was heated to the boil under continuous stirring, followed by filling the bottom tip (1–2 cm) of 1 mL pipettes and allowing to cool to form a salt bridge. 3M KCl was prepared and added to the pipette. Silver wire was immersed in a beaker filled with Mellerud solution for 45 s until the color of the wire changed to black, indicating the presence of AgCl. The prepared AgCl wire was immersed in the KCl solution in the pipette, ensuring no direct contact with the plug at the bottom. The other end of the AgCl wire was connected to the reference electrode output of the three-electrode working cell (lab-made) and secured in place with a plastic cork. Platinum wire was rinsed with ethanol and used as a counter electrode. The buffer solution for lead (II) detection was prepared by mixing 16.64 g of ammonium acetate in 50 mL distilled water, followed by adding ~1100 µL of acetic acid to adjust the pH to 6.0 using a MultiLine P3 pH meter from WTW (Weilheim, Germany). Different concentrations of lead (II) acetate trihydrate (0.2 and 10 mmol·L^−1^) were prepared and the resulting buffer solution was purged with N_2_ for 10 min before commencing the DPSV measurements. A conditioning step at −0.65 V for 1 min was conducted prior to each sweep. A positive voltage scan from −800 mV to 0 mV at 2 mV·s^−1^ rate was passed through the electrodes and the resulting voltammograms were analyzed.

#### 2.2.4. Characterization

Optical microscopic images of the galvanic replacement reaction were captured on a VHX-6000 digital microscope from Keyence (Neu-Isenburg, Germany). The morphology of the galvanically replaced Galinstan droplets and electrodes for initial observation and the exfoliated skin (for droplets only) was studied using a Scios 2 DualBeam (Thermo Fischer Scientific, Dreieich, Germany) SEM at 2 kV. The galvanically replaced droplets and electrodes on the glass substrates were rinsed with distilled water and carefully transferred from the well plate (for droplets) or directly (for electrodes) onto a conductive carbon tape (Science Services, Munich, Germany). The exfoliated skin was carefully separated from the droplet surface and placed on the carbon tape. The area mapping and point scans from EDX were obtained using an Octane Elite EDS System (EDAX, Unterschleissheim, Germany). The area density of Gallium and tungsten was determined by subjecting galvanically replaced Galinstan droplets to an X-ray fluorescence microscope (XRF) of type M4 Tornado from Bruker Nano (Berlin, Germany) under vacuum conditions with a spot size of 20 µm at 50 kV and a 200 µA current. Three spots on each droplet were analyzed for XRF measurement and the resulting mean and standard deviation were used. Surface roughness was measured using a WLI of type NewView 9000 (Zygo, Middlefield, USA), and the resulting data were post-processed using Gwyddion. XPS measurements were conducted using a scanning XPS microprobe of type PHI 5000 VersaProbe III with 1 cm × 1 cm samples of Galinstan and WO-Galinstan electrodes were subjected to an X-ray source at 100 W, with signals obtained at a takeoff angle of 45°. The resulting survey scans and elemental composition were recorded.

## 3. Results and Discussion

### 3.1. Galinstan Electrode Fabrication and Galvanic Replacement

**Figure 1:**
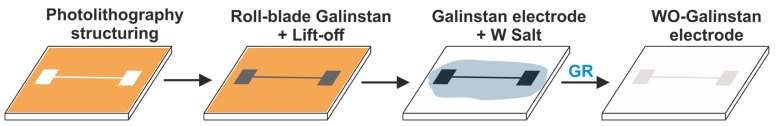
Schematic overview of the photostructuring of Galinstan electrode via lithography followed by galvanic replacement in W salt solution resulting in WO-Galinstan electrode.

**Figure A1:**
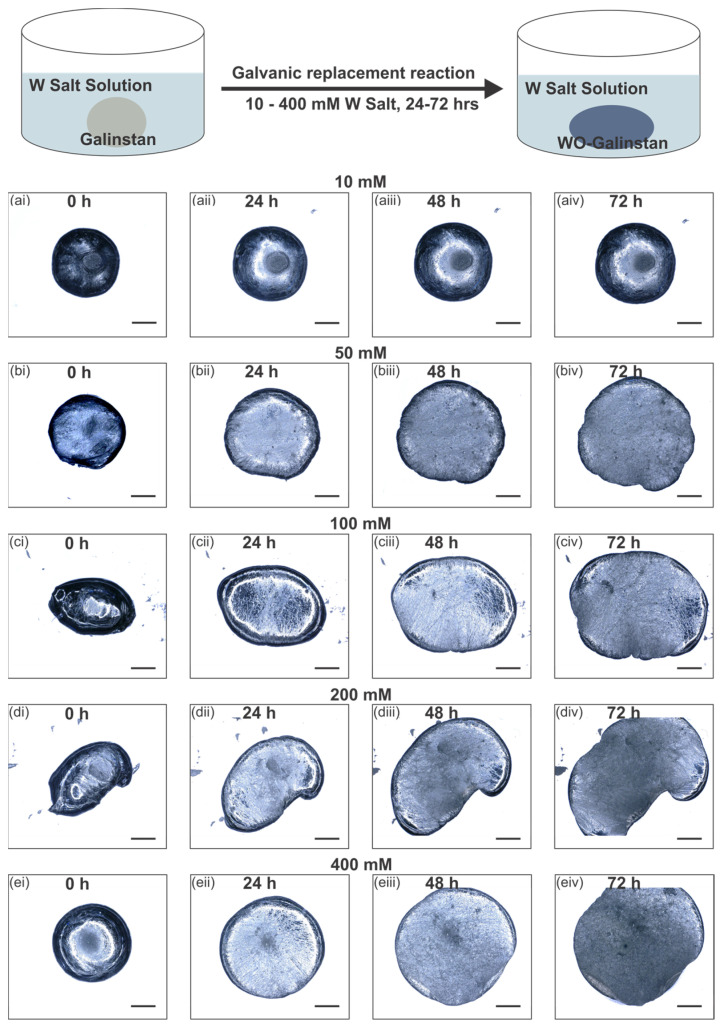
Schematic overview of the galvanic replacement of a 10 µL Galinstan droplet immersed in different concentrations (10, 50, 100, 200, and 400 mM) of 1 mL W salt solution at 0, 24, 48, and 72 h with the corresponding optical microscopy images. (**ai**–**aiv**) No visible change in droplet shape was observed for the 10 mM W salt droplets over 72 h. (**bi**–**eiv**) Definite change from droplet to flattened shape for 50 mM to 400 mM Galinstan droplets indicated successful galvanic replacement. (Scale bar 1 mm).

Galinstan electrodes were fabricated using photolithography, followed by brushing Galinstan on the exposed areas and removal of the remaining photoresist, as shown in the schematic overview in Figure 1. The electrodes were then subjected to GR in 10, 50, 100, 200, and 400 mM W salt solution for 24 h, which was determined by studying the effect of the different concentrations (10, 50, 100, 200, and 400 mM) of W salt on 10 µL of Galinstan droplet for a set period of 24, 48, and 72 h (Figure A1).

**Figure 2:**
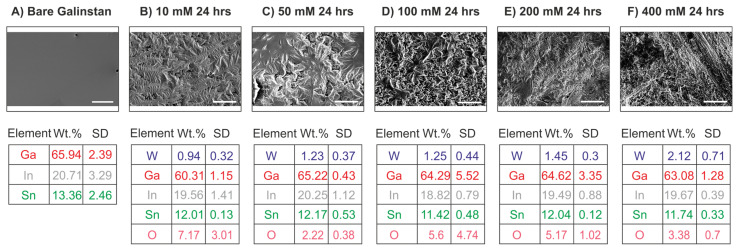
SEM images of an untreated bare Galinstan electrode and a galvanically treated WO-Galinstan electrode in different W salt concentrations for 24 h along with the corresponding EDX spot scan data. (**A**–**F**) Representative EDX spot scan data and the corresponding elemental composition mean in wt.% for four measured spots on each sample with the standard deviation, indicating a gradual increase in the W amount with the highest W amount for the 400 mM W salt-treated Galinstan electrode (Scale bar: 10 µm).

**Figure A2:**
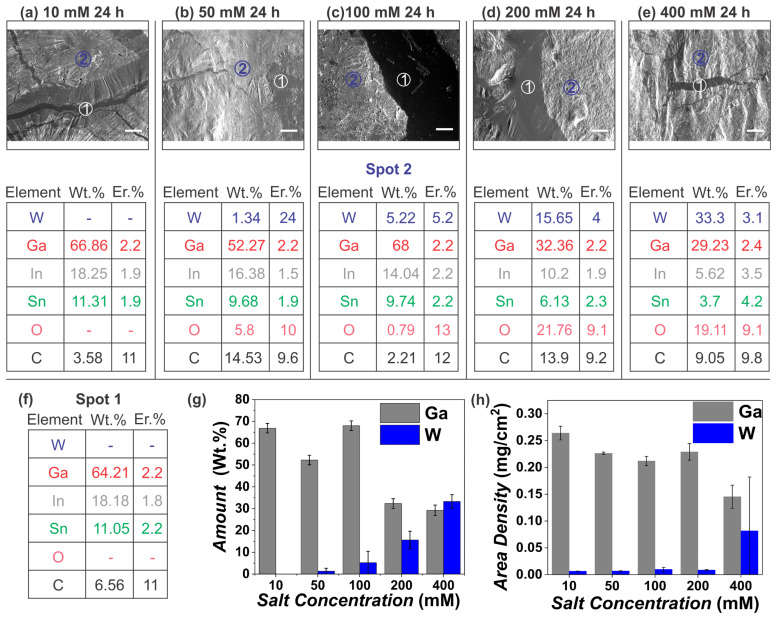
SEM images of Galinstan droplets after 24 h in different concentrations of W salt solutions with supporting EDX spot scan data and XRF measurements. (**a**–**e**) SEM images of Galinstan droplets with the measured spots (1-Galinstan core, 2-W) for EDX and the corresponding elemental concentration in wt. % and error % (Scale bar: 50 µm). (**f**) Representative EDX spot scan data for spot 1 indicated the expulsion of the Galinstan core due to the vacuum conditions inside the SEM. (**g**) Plot derived from EDX spot scan data representing the increase in W amount with increasing salt concentration for 24 h. (**h**) The area density vs. salt concentration plot obtained from XRF measurements for Gallium and Tungsten.

An SEM-EDX elemental spot analysis of the patterned Galinstan electrodes immersed in 10, 50, 100, 200, and 400 mM W salt solution confirmed the presence of tungsten in comparison with the patterned bare Galinstan electrode (Figure 2), with the highest amount detected for the 400 mM sample with 2.12 wt.% tungsten. The respective standard reduction potentials indicate that Galinstan as an alloy of −0.529 V (Ga^3+^/Ga^0^), −0.340 V (In^3+^/In^0^), and −0.138 V (Sn^2+^/Sn^0^) can undergo GR with tungsten −0.119 V (WO^2+^/W^0^), which has a higher reduction potential, although there is not a significantly large difference in their potentials [31,44]. The amount of tungsten increased for the 50 mM electrode compared with the 10 mM electrode (Figure 2B,C); however, it remained almost similar for the 100 mM electrode (Figure 2D). There was a slight increase for the 200 mM electrode, with the maximum tungsten amount for the 400 mM treated sample (Figure 2E,F). For all electrodes, the amount of Ga, In, and Sn was almost constant, with the major fluctuation being the amount of oxygen. While the ratio of tungsten to oxygen was not linear, there was a definite amount of oxygen in excess with the W salt treated electrodes. Moreover, the W salt used for the galvanic replacement reaction has a molecular formula of Na_6_O_39_W_12_·H_2_O, which is 70% tungsten and is commonly used as a precursor to form WO [40]. Interestingly, the overall amount of tungsten for the 400 mM W salt electrode was 10 times less when compared with the GR droplets (Figure A2e). This suggests the significance of the volume of Galinstan, which is much higher for droplets (10 µL) vs. electrodes (~1 µL), allowing a higher amount of tungsten deposition on the liquid metal template depending on the volume. In all the W salt treated electrodes, there was a definite change in the morphology, indicated by the wrinkled surface as seen in the SEM images (Figure 2A–E). To further support GR of Galinstan electrodes treated in different 400 mM W salt, we performed WLI analysis and XPS measurements.

**Figure 3:**
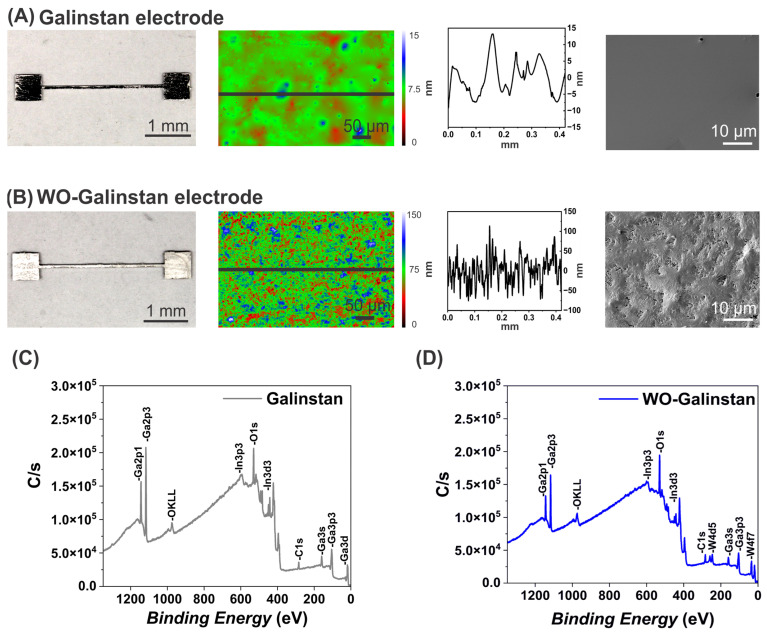
Characterization of Galinstan and WO-Galinstan electrodes. (**A**) Bright-field image of a Galinstan electrode with the surface analysis via WLI alongside the measured profile (right), denoting surface roughness of Ra = 42 nm, supported by the SEM image. (**B**) Bright-field image of a treated WO-Galinstan electrode in 400 mM W salt with the surface analysis via WLI alongside the measured profile (right), denoting surface roughness of Ra = 193 nm, supported by the SEM image clearly indicating a rough surface. (**C**,**D**) Surface composition analysis of untreated Galinstan and treated WO-Galinstan electrodes using XPS, confirming the presence of W with a W4f7 peak.

**Figure A4:**
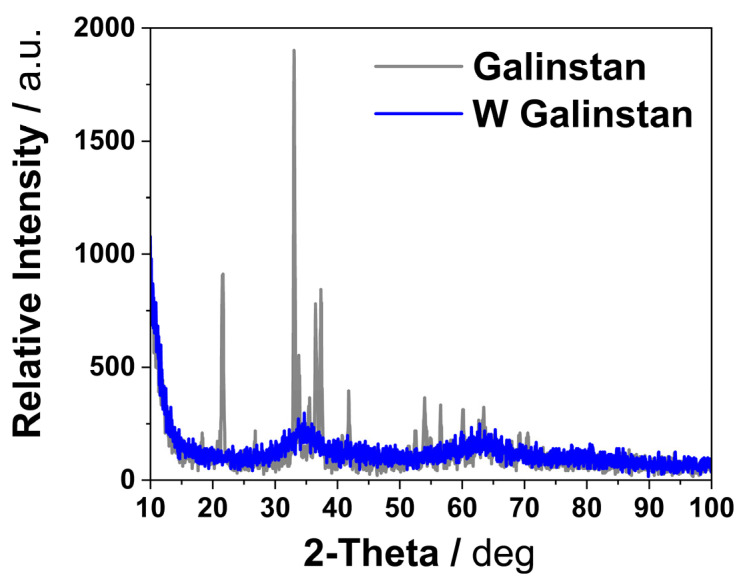
XRD plots for Galinstan powder and 400 mM 24 h treated W-salt Galinstan powder.

The WO-Galinstan electrode was whitish in appearance (Figure 3B) in comparison with the shiny Galinstan pattern (Figure 3A), indicating a definite change post GR. We further characterized the surface roughness of the Galinstan and WO-Galinstan electrodes, fabricated via lithography (Figure 3A,B) using WLI analysis. The Galinstan electrode exhibited a surface roughness Ra of 42 nm, indicating a smooth surface as seen in the SEM image (Figure 3A); whereas the WO-Galinstan electrode had a surface roughness Ra of 193 nm, clearly representing a rough profile and supported by the roughened SEM image (Figure 3B). Furthermore, the Galinstan pattern showed a mean thickness of 1.93 µm ± 0.5 µm, while the thickness of the AZ photoresist was 1.8 µm and the resulting Galinstan pattern post development had taller edges with more uneven areas across the pattern. The extracted surface profile and the WLI image confirmed that some topography exists; however, the overall surface as shown using SEM presented a smooth surface. Overall, the electrode quality looked similar to that reported by Park et al. [45]. On the contrary, the WO-Galinstan electrode had a thickness of 2.68 µm ± 0.8 µm, which is around 0.75 µm greater than the pristine Galinstan electrode, indicating that 24 h immersing in 400 mM W salt resulted in a WO layer with thickness of ~0.75 µm. In addition, we conducted XPS survey scans on Galinstan and WO-Galinstan electrodes and observed that the WO-Galinstan pattern showed a W4f peak at 34 eV in addition to the O1s peak at 530 eV, where a Ga2p1 peak at 1144 eV was observed for the untreated Galinstan pattern (Figure 3C,D). The atomic % data retrieved from the XPS scans further confirmed the presence of 4.78 at. % W4f for the W salt-treated sample, with a ~13 at. % increase in O1s and a ~17 at. % decrease in Ga2p1 in comparison with the untreated sample (Table 1). The SEM-EDX and XPS data for the electrodes and the EDX data for the exfoliated skins, along with the XRD data for droplets (Appendix A), suggest that the resulting WO-Galinstan electrodes are composites of Galinstan (electrode core) and WO (surface), since no LM-WO phases were observed in the XRD plot (Figure A4). This confirms the successful GR of Galinstan to WO-Galinstan electrodes with a definite change in the surface morphology.

### 3.2. Application as a HMI Pb^2+^ Sensor

**Figure 4:**
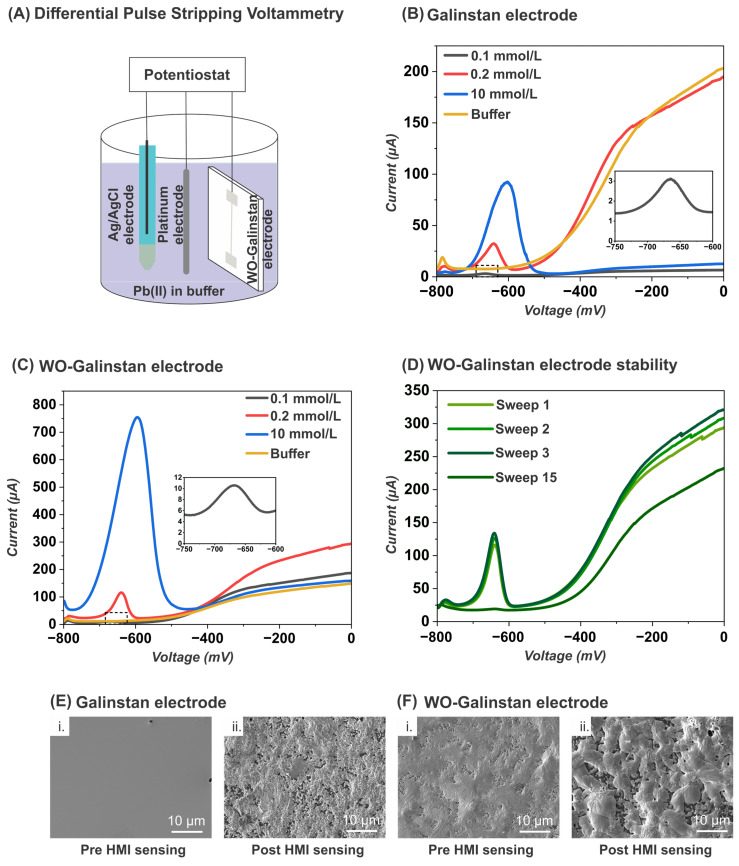
Differential Pulse Stripping Voltammetry (DPSV) experimental setup for Lead (II) detection. (**A**) DPSV setup schematic with the WO-Galinstan electrode as a working electrode, Ag/AgCl as reference electrode, and platinum wire as a counter electrode, with a positive scan of −800 mV to 0 mV at 3 mv·s^−1^. (**B**,**C**) The resulting DPSV voltammograms with the corresponding output current for Lead (II) concentrations of 0.1, 0.2, and 10 mmol·L^−1^ and buffer solution for the Galinstan and WO-Galinstan sensors, respectively. (**D**) Recorded current stability for three consecutive sweeps compared with the fifteenth consecutive sweep for the detection of 0.2 mmol·L^−1^ Pb^2+^ with the WO-Galinstan electrode. (**E**,**F**) SEM images indicated a definite change in the surface morphology of the Galinstan and WO-Galinstan electrodes pre (i) and post HMI (ii) sensing, respectively.

We tested the applicability of the Galinstan and WO-Galinstan electrodes to detect Pb^2+^ HMI in an acetate buffer solution using DPSV (Figure 4A–D). DPSV measurements involve two critical steps. At the first step, the reduction of HMIs occurs by applying a constant cathodic potential to the sensor (in this case at −0.65 V for 60 s), wherein lead ions are pre-concentrated on the sensor surface. In the second step, the applied voltage scan (in this case −800 mV to 0 mV at a 3 mv·s^−1^ scan rate) oxidizes the HMIs, stripping them off the sensor surface [25]. We demonstrate the excellent sensing capability of both the Galinstan and WO-Galinstan electrodes for 10 mmol·L^−1^, 0.2 mmol·L^−1^, and 0.1 mmol·L^−1^ Pb^2+^ in an acetate buffer solution. For the 10 mmol·L^−1^ Pb^2+^ in acetate buffer solution, the WO-Galinstan electrode is about eight times more sensitive than a Galinstan electrode, with a peak current of 754 µA against 92.4 µA for the Galinstan electrode (Figure 4B,C). Both these current magnitudes are significantly higher than the previously reported values on WO-Galinstan marbles, with a similar buffer system reporting a peak current of ~1 µA for the untreated Galinstan marbles, whereas for the WO-coated Galinstan marbles, the peak current was around 80 µA [27]. This observation suggests two things: firstly, the design of the electrode is critical, and a planar electrode allows additional surface area for the lead ions to diffuse into the Galinstan or WO-Galinstan surface, which results in enhanced sensitivity; and secondly, GR of planar electrodes results in a homogeneous WO layer in comparison with the nanopowder coatings. This demonstrates a significant increase in the peak currents and enhanced electrical fields at the Galinstan/WO/Electrolyte interface. In this work, the oxidation peak potential for lead stripping was 600 mV for both Galinstan and WO-Galinstan electrodes in a 10 mmol · L^−1^solution. This potential slightly increases to 640 mV and 660 mV for 0.2 mmol · L^−1^ and 0.1 mmol · L^−1^ Pb^2+^ in an acetate buffer solution. The limit of detection for both the Galinstan and WO-Galinstan electrodes was 0.1 mmol · L^−1^ Pb^2+^, presenting three times higher sensitivity for the WO-Galinstan electrodes (Current output ~10 µA) in comparison with the Galinstan electrodes (Current output ~3 µA). We also demonstrated the stability of the WO-Galinstan sensor for successive DPSV sweeps in 0.2 mmol·L^−1^ at 3 mv·s^−1^ with identical voltammograms and minor differences in the output currents (Sweep 1: 116 µA, Sweep 2: 126 µA, Sweep 3: 134 µA; Figure 4D). Interestingly, the output current dropped to 19 µA for Sweep 15, which suggests that 15 consecutive sweeps (01:06 h) was the limit of the fabricated WO-Galinstan electrodes. However, the pre-concentration step was only implemented once before subjecting the WO-Galinstan electrode to 15 consecutive sweeps, which explains the drop in the output current. Furthermore, we observed a morphology change on both the electrode surfaces after HMI sensing (Figure 4E,F), which suggests that the lead ions amalgamated with the Galinstan and WO-Galinstan electrodes during the pre-concentration step. The performance of the Galinstan and WO-Galinstan electrodes for Pb^2+^ in comparison with other reported works is presented in Table 2. While the limit of detection values depend on the measurement technique and type of electrode (higher volumes for hanging droplet electrodes), the Galinstan and GR WO-Galinstan electrodes outperformed the WO_3_-coated Galinstan droplets. We believe that increasing the thickness of the Galinstan and WO-Galinstan electrodes would enable much lower detection limits.

## 4. Conclusions

In summary, this study presented, for the first time, structured Galinstan and WO-Galinstan electrodes via photolithography and galvanic replacement using a W salt. We first examined the effect of different W salt concentrations of 10, 50, 100, 200, and 400 mM on patterned Galinstan electrodes via GR over a period of 24 h using SEM-EDX. Further characterization via WLI, XPS, and XRD confirmed the successful WO coating on Galinstan electrodes, at the optimal concentration of 400 mM W salt for 24 h. Both Galinstan and WO-Galinstan electrodes demonstrated excellent sensitivity and detection stability for 10, 0.2, and 0.1 mmol·L^−1^ Pb^2+^ HMI in an acetate buffer solution, with a 0.1 mmol·L^−1^ Pb^2+^ limit of detection. Crucially, the WO-Galinstan sensor is about three times more sensitive than the currently reported Galinstan sensors for 0.1 mmol·L^−1^ Pb^2+^, confirming the importance of WO in enhancing the electrical field at the WO-Galinstan-lead buffer interface. We believe that this work will allow the applicability of GR to planar geometries, like electrodes in sensors, and the implementation of WO-Galinstan sensors for Pb^2+^ detection in a toxic-free environment, for further application in microfluidics and MEMS.

## Figures and Tables

**Table 1:** Atomic % of the recorded XPS scans for untreated Galinstan and galvanically treated WO-Galinstan.

	O1s (at. %)	Ga2p1 (at. %)	Ga3d (at. %)	W4f (at. %)
Galinstan	64.62	33.63	1.75	0
WO-Galinstan	77.26	17.96	0	4.78

**Table 2:** Performance comparison of reported Gallium liquid metal sensors for Pb^2+^ detection with their corresponding measurement techniques, limit of detection values, and appropriate comments.

Materials	Measurement Technique	Limit of Detection (mmol·L^−1^)	Comments	Reference
Galinstan (Hanging droplet)	Differential Pulse Voltammetry	0.91	Hanging droplets offer higher LM volume for HMI detection	[23]
Galinstan (Hanging droplet)	Differential Pulse Stripping Voltammetry	0.008	In addition to a higher volume, stripping voltammetry involves a pre-conditioning step wherein lead ions amalgamate, resulting in lower limit of detection values	[24]
Galinstan with WO_3_ coating (Hanging droplet)	Differential Pulse Stripping Voltammetry	10	WO_3_ micro-nanopowder coatings on Galinstan droplets	[27]
EGaIn-GO particles suspended on carbon paper	Differential Pulse Voltammetry	10	EGaIn-GO particles fabricated via sonication of EGaIn in GO suspension	[29]
Galinstan and WO-Galinstan planar electrodes via GR	Differential Pulse Stripping Voltammetry	0.1	Both Galinstan and WO-Galinstan (via GR) planar electrodes show excellent sensitivity to 0.1 mmol·L^−1^ Pb^2+^	This Work

**Figure A3:**
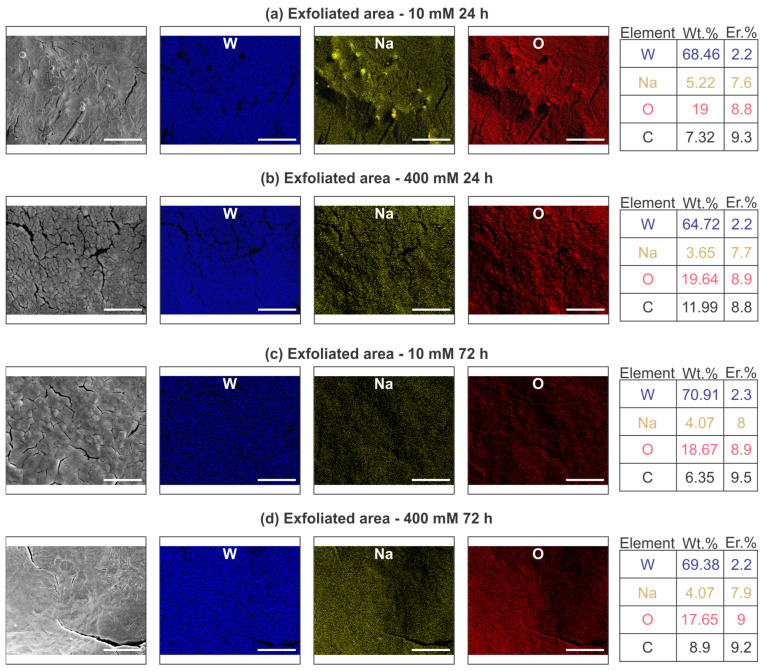
SEM-EDX mapping images for the exfoliated skin of Galvanically replaced Galinstan droplets in 10 mM and 400 mM for 24 h (**a**,**b**) and 72 h (**c**,**d**), respectively, with the corresponding element weight.%. Tungsten amount for all four exfoliated skins was similar ~70 wt.% with ~20 wt.% Oxygen. (Scale bar: 20 µm).

## Data Availability

Data are contained within the article or Appendix A.

## Appendix A

### Appendix A.1. Galvanic Replacement on Galinstan Droplets

No visible changes were observed for 10 mM W salt concentration even after 72 h, suggesting the need for higher salt concentrations. The captured images clearly showed an incremental change in the droplet size from the initial shape, especially for W salt concentrations of 50 mM and higher with increasing time. A transition from a droplet shape to a flattened shape was observed from 24 to 72 h, indicating a definite change in the morphology and appearance of pristine Galinstan droplets post GR. At 72 h, the droplet completely lost its shape and flattened to the maximum limit for concentrations exceeding 50 mM W salt (Figure A1biv,civ,div,eiv). We performed an SEM-EDX elemental spot analysis for Galinstan droplets immersed in 10, 50, 100, 200, and 400 mM W salt for 24 h, and investigated the findings as seen in Figure A2a–e. Two spots were analyzed for each droplet, with spot 1 (Figure A2f) clearly indicating the liquid metal core exuding out from the core under SEM vacuum conditions, with 64.21 wt.% Ga, 18.18 wt.% In, and 11.05 wt.% Sn. This suggests that GR occurred at the droplet surface and the core remained unaffected at least for the 24 h reaction period. For 10 mM W salt, an EDX spot scan (Figure A2a) indicated the absence of tungsten, with the major constituents being that of Galinstan; whereas for the 50 mM W salt sample, 1.34 wt.% tungsten existed with an error of 24% (± 0.32 wt.%), suggesting poor GR. A significant increase in tungsten was observed for 100 mM (error ± 0.27 wt.%) and 200 mM (error ± 0.62 wt.%) W salt (Figure A2c,d) with a circa 5% error compared with the 50 mM sample. The EDX data indicate that with increasing tungsten amount, the corresponding gallium, indium, and tin amounts decreased, which confirms that GR is dominant for the elements with the maximum difference in their reduction potentials, i.e., gallium, indium, and tin against tungsten. However, some discrepancies were observed in the case of the 50 and 100 mM W salt samples, where the gallium amount increased along with tungsten (Figure A2b,c), with similar indium and tin amounts. This could possibly be attributed to the lower amount of tungsten post GR, which resulted in a high variability in the obtained EDX data. For the 400 mM W salt sample (Figure A2e), the highest amount of 33.3 wt.% tungsten was observed along with 29.23 wt.% gallium, 5.62 wt.% indium, and 3.7 wt.% tin. Interestingly, for the 200 and 400 mM W salt droplets, ~20 wt.% oxygen was present alongside tungsten, implying the formation of WO. This is plausible since the 10 mM W salt droplet showed neither tungsten nor oxygen, whereas for the Galinstan droplets treated in 50 and 100 mM W salt (and higher), both tungsten and oxygen were present. The data obtained from the EDX spot scan for gallium and tungsten plotted for different W salt concentrations showed a definite trend of increasing tungsten amount compensated by the decreasing gallium amount, as seen Figure A2g. An area density versus salt concentration plot obtained using XRF (Figure A2h) showed a slightly similar trend, with the highest tungsten area density for a 400 mM W salt concentration, with a high standard deviation value indicating a difference in the tungsten area density at different spots. This deviation could be attributed to the transfer of the treated Galinstan droplets from the well plate to a glass substrate for XRF measurements using a pipette tip, which unintentionally affected the droplet shape. Both EDX and XRF data indicate a definite presence of tungsten and the highest amount for 400 mM W salt, confirming galvanic replacement. In conclusion, 24 h immersion of a Galinstan droplet in 400 mM W salt solution showed a significant change in the droplet appearance and morphology, confirmed by the presence of tungsten via EDX and XRF measurements. Interestingly, the retrieved exfoliated tungsten oxide skin from the droplet surface for the 10 mM and 400 mM W salt samples at 24 and 72 h showed almost identical wt.% of tungsten (around 70 wt.%) and oxygen (around 19 wt.%), again implying the formation of WO (Figure A3). The EDX mapping of the exfoliated skins complimented the XRF measurements in Figure A2h, where tungsten was detected for both the 10 mM and 400 mM W salt droplets. The skin morphology was consistent for all exfoliated skins with cracks possibly from salt dehydration. Around 4 wt.% sodium was detected, which was most probably from the inner layers and not the surface of the skin since the droplet surface was rinsed with distilled water before conducting the SEM-EDX measurements. This implies that a 24 h reaction time was sufficient for GR to take place and longer times are avoidable. To confirm that GR occurred, we analyzed Galinstan and 400 mM W salt-treated Galinstan powders using XRD. The resulting XRD plots showed sharp peaks for GaOOH (Gallium oxide hydroxide; PDF 00-054-0910) at 21°, 34°, 38°, and 42°, along with an Indium Tin alloy (PDF 04-004-7736) with a major peak at 33° and some small peaks at 37°, 53°, 57°, and 64° (see Figure A4). Interestingly, the W salt Galinstan powder did not show any sharp peaks, with two low broad peaks at 34° and 63°, indicating that the GaOOH and Indium/Tin alloy had a loss in intensity and possibly crystallinity. This suggests that there was an obvious change post GR, possibly representing an amorphous form of WO. These results confirmed that 24 h treatment in 400 mM W salt is optimal for successful GR.

### Appendix A.2. Sample Preparation for XRD Measurements

50 µL Galinstan was sonicated in 1 mL ethanol and 1 mL 400 mM W salt solution respectively for 30 min at 60% amplitude using a sonotrode (UP200St, Hielscher Ultrasonics, Teltow, Germany). The W-salt Galinstan sonicated mixture was left to react for 24 h. The W-salt Galinstan mixture was passed through a filter paper (type 601P) and washed with distilled water to get rid of excess tungsten and sodium residues, followed by drying at room temperature overnight. For the Galinstan ethanol mixture, the supernatant was removed and the remaining powder was left to dry at room temperature overnight. The X-ray diffraction (XRD) patterns of these powders were recorded in a 2-Θ range of 10–100° with a step size of 0.05° using a D8 DISCOVER Diffractometer (Bruker, Karlsruhe, Germany).
